# Use of Patient Portal Messaging and Self-Reported Copays Among US Adults 50 Years or Older

**DOI:** 10.1001/jamahealthforum.2025.0168

**Published:** 2025-04-04

**Authors:** Terrence Liu, Matthias Kirch, Erica Solway, Dianne C. Singer, J. Scott Roberts, Jeffrey T. Kullgren, Tammy Chang

**Affiliations:** 1 Institute for Healthcare Policy and Innovation, University of Michigan, Ann Arbor; 2 Veterans Affairs Center for Clinical Management Research (CCMR), Veterans Affairs Ann Arbor Healthcare System, Ann Arbor, Michigan; 3 Department of Internal Medicine, University of Michigan Medical School, Ann Arbor; 4 Department of Health Behavior and Health Equity, University of Michigan School of Public Health, Ann Arbor; 5 Department of Health Management and Policy, University of Michigan School of Public Health, Ann Arbor; 6 Department of Family Medicine, University of Michigan Medical School, Ann Arbor

## Abstract

This survey study characterizes the prevalence of reported copays for portal messages by health insurance type among US adults aged 50 years or older who used portal messaging.

## Introduction

Secure portal messages allow patients to connect with their health care teams between clinic visits. Older adults, who often have multiple chronic conditions, may particularly benefit from portal messages, given challenges with accessing care and addressing all health issues comprehensively in a single office visit. In 2020, telemedicine reimbursement expanded, allowing clinicians to bill for portal messages that require clinician time for medical decision-making.^1,2^ However, copays associated with portal messages may discourage patients, especially individuals with low income, from communicating important health information through the portal. Furthermore, coverage for billed portal messages vary by insurance plan. Our objective was to characterize the prevalence of reported copays for portal messages by health insurance type among US adults aged 50 years or older who used portal messaging.

## Methods

From February 22 to March 12, 2024, we conducted a cross-sectional survey using a probability-based sample of US adults aged 50 years or older (43.7% completion rate [eMethods in Supplement 1]) through the University of Michigan National Poll on Healthy Aging and weighted the sample to reflect population figures from the US Census Bureau.^3^ The University of Michigan institutional review board deemed this study exempt and waived informed consent because the data were deidentified. We followed the AAPOR reporting guideline.

Older adults were asked if they had a patient portal, had messaged a health care professional (the term *health care provider* was used in our survey) through the portal, and whether they were charged a copay for sending a message. Self-reported sociodemographic information and health insurance information were collected. We used multivariable logistic regression with average marginal effects to estimate associations between having been charged a copay for sending a portal message (primary outcome) and health insurance type (main variable; eMethods in Supplement 1). We used sample weights to account for probability-based sampling design and survey nonresponse. Two-sided *P* < .05 was considered statistically significant. Analyses were conducted using Stata, version 18.

## Results

Of the 3212 respondents (mean age, 65 [95% CI, 64-66] years; weighted, 53.3% women and weighted, 46.7% men), 76.2% (95% CI, 72.9%-79.1%) of respondents reported having a patient portal and 64.7% (95% CI, 62.3%-67.2%) reported sending a portal message in the past year (Table). Across health insurance types, most older adults reported having sent a portal message in the past year, ranging from 58.6% (95% CI, 44.3%-72.8%) of those with traditional Medicare without supplemental insurance to 75.2% (95% CI, 67.9%-82.5%) of those with Veterans Affairs coverage (Figure). A lower proportion of older adults (13.1% [95% CI, 11.4%-15.0%]) reported being a charged a copay for sending a portal message (Table), with the highest estimate being 16.6% (95% CI, 11.2%-21.9%) for those privately insured, followed by 15.9% (95% CI, 4.5%-27.4%) for those dual-eligible or with Medicaid (Figure).

**Table.** Estimated Characteristics of US Adults Aged 50 Years or Older

Characteristic	Respondents, No. (n = 3212)	Weighted % (95% CI)
Having a patient portal^a^	2478	76.2 (72.9-79.1)
Sent a portal message^b^	1628	64.7 (62.3-67.2)
Charged copays for a portal message^c^	180	13.1 (11.4-15.0)
Age, y		
50-64	1482	53.2 (47.8-58.6)
65-101	1730	46.8 (41.4-52.2)
Gender identity		
Male	1436	46.7 (43.4-50.0)
Female	1756	53.3 (49.9-56.6)
Race and ethnicity		
Hispanic	414	11.1 (9.6-12.8)
Non-Hispanic Black	597	10.2 (9.3-11.1)
Non-Hispanic White	2067	70.6 (68.9-72.3)
Non-Hispanic other^d^	134	8.1 (6.9-9.4)
Educational level		
High school or lower	672	39.9 (36.9-42.9)
Some college	1334	28.1 (26.9-29.4)
Bachelor’s degree or higher	1206	32.1 (28.9-35.4)
Annual household income, $		
<60 000	1546	48.4 (46.9-50.0)
≥60 000	1666	51.6 (49.9-53.2)
Living in an MSA		
Yes	2724	85.1 (82.3-87.5)
No	488	14.9 (12.5-17.7)
Having broadband internet at home		
Yes	2900	89.9 (88.1-91.4)
No	312	10.1 (8.6-11.9)
Past-year health care utilization^e^		
0	212	7.7 (6.5-9.1)
1-2	1033	33.2 (31.6-34.8)
≥3	1949	59.1 (57.4-60.8)
Self-rated physical health		
Excellent, very good, or good	2586	81.1 (79.2-82.8)
Fair or poor	620	18.9 (17.2-20.8)
Self-rated mental health		
Excellent, very good, or good	2814	89.2 (88.2-90.3)
Fair or poor	333	10.7 (9.7-11.8)
Health insurance type		
Private^f^	1141	40.1 (36.5-43.8)
Medicare Advantage	855	23.5 (20.4-26.9)
Traditional Medicare with supplemental insurance	522	15.4 (13.6-17.3)
Traditional Medicare without supplemental insurance	142	4.2 (3.5-5.1)
Dual-eligible or only Medicaid^g^	310	9.6 (8.0-11.5)
VA or military^h^	242	7.3 (6.6-8.0)

Abbreviations: MSA, metropolitan statistical area; VA, Veterans Affairs.

^a^ Answering yes to having a patient portal, defined as a personal, password-protected connection to a health care practice for exchanging information through a computer, telephone, or tablet.

^b^ Answering yes to the question, “In the past year, have you sent a message to a health care provider using an online patient portal?”

^c^ Answering yes to the question, “Have you ever paid a copayment or been charged money for exchanging messages with your health care provider over the patient portal?”

^d^ Asian, American Indian or Alaska Native, Native Hawaiian or Pacific Islander, more than 1 race, and different race.

^e^ Answering the survey question: “In the past year, about how many times did you get medical care from a health care provider (in person or virtually)?”

^f^ Includes respondents selecting retiree health plans, employer-sponsored insurance, or individual insurance plans purchased directly by individuals including from an online marketplace.

^g^ Includes respondents selecting Medicaid as their only insurance or Medicaid in addition to another type of insurance.

^h^ Includes respondents selecting VA and TRICARE coverage. This category includes respondents who reported having VA or TRICARE coverage in addition to another type of insurance.

**Figure.**
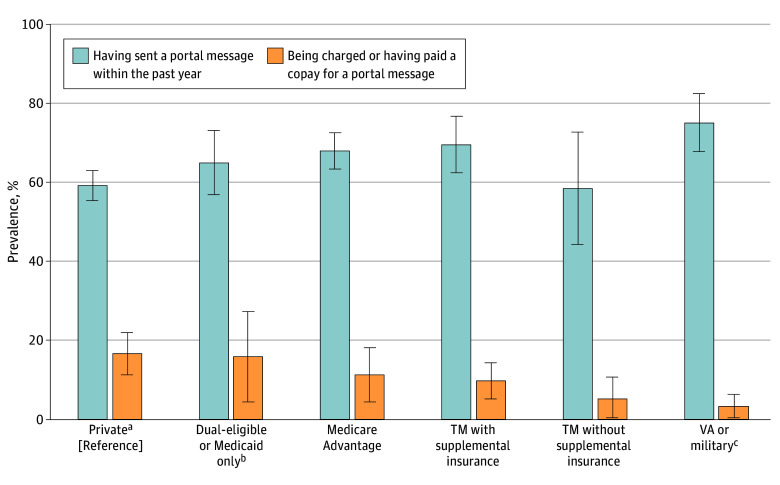
Self-Reported Use of Portal Messages and Charged Copays by Health Insurance The adjusted estimated prevalence of having sent a portal message in the past year and being charged or having paid a copay for a portal message is shown. The models were adjusted for age, gender identity, race and ethnicity, educational level, income, living in a metropolitan statistical area, having home internet, number of times receiving medical care from a health care professional in the past year, and self-reported health status. Private health insurance was used as the reference group for analyses. Error bars indicate 95% CIs. TM indicates traditional Medicare; VA, Veterans Affairs. ^a^Includes respondents selecting retiree health plans, employer-sponsored insurance, or individual insurance plans purchased directly by individuals including from an online marketplace. ^b^Includes respondents selecting Medicaid as their only insurance, or Medicaid in addition to another type of insurance. ^c^Includes respondents selecting VA and TRICARE coverage. This category includes respondents who reported having VA or TRICARE coverage in addition to another type of insurance.

## Discussion

In this nationally representative survey study of adults aged 50 years or older, most individuals reported sending a portal message to their health care professional in the past year, with a minority reporting ever having a copay for such messages. The low prevalence of copays in our study is consistent with prior studies showing full coverage of portal message out-of-pocket costs among privately insured patients over 80% of the time, with estimates ranging from $14 to $40 per message when not covered.^4^ We found that Medicaid-eligible individuals had similar estimated prevalences of reported copays compared with privately insured individuals. Although we lack data on self-reported out-of-pocket costs, our findings highlight a potential gap where Medicaid-eligible individuals may not experience the same degree of cost-sharing reduction they typically receive for many other clinical services.^5^ Limitations include inability to identify nonbillable messages and data being self-reported and subject to possible recall bias, which may underestimate the true prevalence of charged copays.^6^ Future research should explore how variation in portal message copays across payers, particularly Medicaid, impact health care utilization among individuals with low income.

## Notes

Supplement 1.
eMethods.

Supplement 2.
Data Sharing Statement
