# Supplementary material for: Use of Patient Portal Messaging and Self-Reported Copays Among US Adults 50 Years or Older
Source: JAMA Health Forum. 2025 Apr 4;6(4):e250168. doi: 10.1001/jamahealthforum.2025.0168 (PMC11971665; doi:10.1001/jamahealthforum.2025.0168)
Supplement: Supplement 1. — eMethods. [file jamahealthforum-e250168-s001.pdf]

Supplementary Online Content

Liu T, Kirch M, Solway E, et al. Use of patient portal messaging and self-reported copays among US adults 50 years or older. *JAMA Health Forum.* 2025;6(4):e250168. doi:10.1001/jamahealthforum.2025.0168

eMethods.

This supplementary material has been provided by the authors to give readers additional information about their work.

© 2025 Liu T et al. *JAMA Health Forum.*

eMethods.

Survey Methodology

The University of Michigan National Poll on Healthy Aging (NPHA) is a recurring, nationally representative household survey of adults aged 50 and older on health-related issues. The NPHA is directed by the University of Michigan Institute for Healthcare Policy and Innovation and is sponsored by AARP and Michigan Medicine, the University of Michigan's academic medical center.

By tapping into the perspectives of older adults and their caregivers, the NPHA helps inform the public, health care providers, policymakers, and advocates on issues related to health, health care, and health policy affecting U.S. adults aged 50 and older and their families. NPHA results are shared widely to elevate the voices of the public, inspire future research, and inform national dialogue.

NORC AmeriSpeak® panel

NPHA surveys are conducted using NORC's AmeriSpeak® panel, a nationally representative probability-sample panel of U.S. households. A general population sample of U.S. adults aged 50 and older was selected from NORC's AmeriSpeak Panel for this study. Sample was selected to reach 2,000 interviews split evenly between 50 to 64 and 65+ age groups. Sampling was restricted to active AmeriSpeak panel members. More specifically, a respondent needs to have responded to at least one survey within the prior six months to be selected. The sample for a specific study is selected from the AmeriSpeak Panel using sampling strata based on age, race/Hispanic ethnicity, education, and gender (24 sampling strata in total). Sample selection takes into account the expected differential survey completion rates across the sampling strata. The AmeriSpeak® panel itself is a randomly selected US household sample using area probability and address-based sampling. Surveys can be conducted via internet or phone. Data are weighted to match the most recent Current Population Survey (CPS).

The February 2024 NPHA survey was sampled using both NORC's core sample, which consists of frequent responders to surveys, and an oversample based on race and ethnicity to increase the diversity of respondents and improve our ability to understand subgroup differences in key outcomes. Recruiting from the oversample resulted in a decrease in survey completion rates, from 71.0% for the core sample, to 43.7% for the full sample including oversample. For this analysis, we decided to include the results from the oversample to examine differences in experiences of patient portal messages by insurance type. The oversample increased the numbers of respondents with each insurance type, but especially improved the precision of estimates for those who are dual-eligible or on Medicaid only, and those with VA insurance.

Health Insurance Survey Item

In the survey, respondents were asked the question, "What type(s) of health insurance do you have now?". Responses options included:

1. Traditional Medicare without a Medigap plan
2. Medicare Advantage plan
3. Traditional Medicare plus Medigap (a supplemental Medicare plan)
4. Medicaid
5. Retiree health plan from a job that you or someone else retired from
6. Insurance provided through your own or someone else's employer
7. Individual insurance plan you bought directly, including from an online marketplace
8. VA / CHAMPVA
9. Military health care (TRICARE)
10. Other (please specify): [TEXTBOX]
11. None – no health insurance of any kind

© 2025 Liu T et al. *JAMA Health Forum.*

Of 3,379 respondents to this survey item, 71 selected “None - no health insurance of any kind” and were excluded from analysis. Free-text responses from “Other (please specify)” were re-coded into survey response items when appropriate (e.g. “Medi-Cal” to “Medicaid”, or “Employer” to “Insurance provided through your own or some else’s employer”). Free-text responses that were ambiguous were not re-coded. Individuals with free-text responses that were not re-coded into an existing category were excluded from analyses. 8 respondents with missing data were also excluded.

Of 3,300 respondents who reported having a form of health insurance, most respondents selected only one option (2,573, 78%). Among respondents who selected ≥2 options for health insurance, 611 (19%) selected two options, 96 (3%) selected three options, 14 (0.4%) selected four options, and 6 (0.2%) selected five options. Of the 3,300 respondents with free-text responses unable to be re-coded into existing categories, we excluded 88 individuals from our analysis.

For respondents who had selected ≥2 options for health insurance for the survey item, we developed criteria that allowed for categorization into 6 mutually exclusive categories. Respondents who had selected “VA/CHAMPVA” or “Military health care (TRICARE)”, in addition to another insurance option, were categorized as “VA or military” in our main analysis. Respondents who had selected Medicaid in addition to another insurance option were categorized as “Dual-eligible or only Medicaid” in our main analysis. Respondents who had selected “Traditional Medicare without a Medigap plan” in addition to another insurance option were categorized as “Traditional Medicare with supplemental insurance”. Respondents who had selected “Traditional Medicare with supplemental insurance” and “Private” insurance were categorized as “Traditional Medicare with supplemental insurance”.

The 6 mutually exclusive categories for health insurance type used in our main analysis are summarized below:

**Health Insurance Type**	**Criteria**
Private	Respondents selecting retiree health plans, employer-sponsored insurance, or individual insurance plans purchased directly by individuals including from an online marketplace
Medicare Advantage	Respondents selecting Medicare Advantage plan
Traditional Medicare with supplemental insurance	Respondents selecting Traditional Medicare plus Medigap (a supplemental Medicare plan)
Traditional Medicare without supplemental insurance	Respondents selecting Traditional Medicare without a Medigap plan
Dual-eligible or only Medicaid	Respondents selecting Medicaid as their only insurance, or Medicaid in addition to another type of insurance.
VA or military	Respondents selecting VA/CHAMPVA and military health care (TRICARE) coverage. This category includes respondents who reported having VA or TRICARE coverage in addition to another type of insurance.

© 2025 Liu T et al. *JAMA Health Forum.*
