# Supplementary material for: Use of Patient Portal Messaging and Self-Reported Copays Among US Adults 50 Years or Older
Source: JAMA Health Forum. 2025 Apr 4;6(4):e250168. doi: 10.1001/jamahealthforum.2025.0168 (PMC11971665; doi:10.1001/jamahealthforum.2025.0168)
Supplement: Supplement 2. — Data Sharing Statement [file jamahealthforum-e250168-s002.pdf]

Data Sharing Statement

Liu. Use of Patient Portal Messaging and Self-Reported Copays Among US Adults 50 Years or Older. *JAMA Health Forum*. Published April 04, 2025. doi:10.1001/jamahealthforum.2025.0168

Data
Data available: Yes
Data types: Deidentified participant data
How to access data: Deidentified survey data will be made available in the future at: https://www.healthyagingpoll.org/reports-more/data
When available: beginning date: 01-01-2026

Supporting Documents
Document types: Other (please specify)
Additional Information: Codebook, survey instrument
How to access documents: https://www.healthyagingpoll.org/reports-more/data
When available: beginning date: 01-01-2026

Additional Information
Who can access the data: Anyone requesting the data
Types of analyses: For any purpose
Mechanisms of data availability: Data will be made publicly available without investigator support
